# Application of calibrated forceps for assessing mechanical nociception with high time resolution in mice

**DOI:** 10.1371/journal.pone.0172461

**Published:** 2017-02-17

**Authors:** Hideki Kashiwadani, Yuichi Kanmura, Tomoyuki Kuwaki

**Affiliations:** 1 Department of Physiology, Graduate School of Medical and Dental Sciences, Kagoshima University, Kagoshima, Japan; 2 Department of Anesthesiology, Graduate School of Medical and Dental Sciences, Kagoshima University, Kagoshima, Japan; Yeshiva University Albert Einstein College of Medicine, UNITED STATES

## Abstract

In order to investigate the basic physiological mechanisms of pain and the anti-nociceptive effects of analgesics, development of pain assays in mice is critical due to the advances of genetic manipulation techniques. The von Frey hairs/Semmes-Weinstein monofilaments test (von Frey test) has long been applied to examine mechanical nociception in mice. Though the von Frey test is a well-established and standardized method, it is inappropriate to assess a rapid change in the nociceptive threshold because voluntary resting/sleeping states are necessary to examine the response. In this study, we assessed the effectiveness of calibrated forceps to determine the mechanical nociceptive threshold in mice. Repeated daily measurements of the threshold over 5 days indicated that the device obtained stable and reliable values. Furthermore, repeated measurements with 5 minute intervals revealed that the device detected the rapid change of the threshold induced by remifentanil, a short-acting μ-receptor agonist. These results indicate that the calibrated forceps are well-suited for measuring the mechanical nociceptive threshold in mice, and are useful in assessing the effects of short-acting analgesics on mechanical nociception.

## Introduction

In clinical anesthesia, short-acting analgesics are gaining importance recently because they allow for quick and on-demand control of the depth of anesthesia and enable swift recovery. However, the basic research of short-acting analgesics for mechanical pain was only limited and the evaluation was mainly performed for thermal pain because the established protocols for assessing the mechanical nociception has low time resolution (>30 minutes). Therefore the new techniques for mechanical nociception with high time resolution (< 5minutes) has been required.

When investigating the mechanisms of nociception and the effects of anti-nociceptive drugs pre-clinically, some of the most common techniques include determining the threshold and measuring the duration of behavioral responses to nociceptive stimulation in rodents [1]. Nociceptive tests have long been performed mainly in rat. However, the mouse models of nociception have recently increased due to the development of genetic engineering techniques in mice [2].

Distinct populations of peripheral nociceptors and central nociceptive neurons are involved in nociceptive information processing depending on the nociceptive modality [3], so it is important to evaluate the effects of analgesics respectively to those modalities. Though there are various types of nociceptive tests, most are classified into three modality categories: thermal, chemical, and mechanical [4]. For investigating the thermal nociceptive threshold, the tail flick test [5], the hot plate test [6, 7], and the plantar test [8] are well developed, and for investigating chemical nociception, the formalin test is most common [9, 10]. The nociceptive tests for thermal and chemical stimulation are well characterized and standardized in both rats and mice [4].

The von Frey hairs/Semmes-Weinstein monofilaments test (von Frey test) and the Randall-Selitto test are commonly used to examine mechanical nociception in both mice and rats. The von Frey test consists of filaments made of plastic fibers that exert a calibrated pressure depending on their diameter [11]. To determine the nociceptive threshold using the von Frey test, repeated measurements using several filaments with different diameters (thus different calibrated pressures) are necessary [11]. However, although the mouse is unrestrained, the voluntary change in behavioral states during measurements cannot be controlled even though they significantly affect the threshold [12]. As a result, the von Frey test requires an extended experimental duration and is inappropriate for examining a rapid change in the nociceptive threshold, even though the von Frey test is superior in terms of the restraint-free method. Electronic von Frey devices that reduce the necessary time for determining the threshold have been developed, but the demand for voluntary resting/sleeping states remains an issue. In the Randall-Selitto test, the hind paw of a rat is placed on a stable base and a controlled pressure is applied with a blunt point [13]. Though the test gives stable results [14], it demands a high proficiency in behavioral experimentation and a large number of animals [4]. Moreover, the subject is restrained in a vertical, unnatural position in order to place its hind paw on the apparatus, so extensive acclimatization is required to obtain reliable values [14, 15]. These two tests measure mechanical nociception at fairly low time resolution, so there remains a need to develop new techniques that can measure at a higher resolution (< 5 minutes).

Recently, as an alternate procedure to the Randall-Selitto test, a new algometer using calibrated forceps has been developed [16–18]. Luis-Delgado and her colleagues reported that the calibrated forceps could measure the mechanical threshold more reliably, easily, quickly, reproducibly, and more reliably than the classical von Frey test in rats [16]. Furthermore, several researchers have recently reported a steady-state mechanical nociceptive threshold using the device [19–21]. Thus we hypothesized that we could use calibrated forceps to detect a rapid change in the mechanical nociceptive threshold of mice caused a short-acting analgesic with high time resolution.

In this study, we measured the behavioral response threshold to noxious mechanical stimuli using calibrated forceps on the tails of mice. The data indicated that the device gave reliable and stable values for the threshold. Furthermore, the device detected the rapid change in the threshold induced by subcutaneous injection of remifentanil, a short-acting analgesic [22, 23]. These results suggest that the calibrated forceps may be useful to assess the relatively quick (within an hour) change of the nociceptive threshold in mice.

## Materials and methods

### Animals

Male C57BL/6 mice (28.0 to 39.0 g, n = 36) were originally purchased from the Kyudo Company (Tosu, Saga, Japan). Mice were housed in an environment maintained at 25 ± 1°C with free access to food and water under a 12 h light-dark cycle with lights on from 7:00 to 19:00. Experiments were performed between 14:00 and 19:00. Mice were euthanized after the behavioral test session by cervical dislocation under deep urethane anesthesia. All experiments were performed in accordance with The Physiological Society of Japan’s guidelines and were approved by the Experimental Animal Research Committee of Kagoshima University.

If not otherwise specified, all animals were acclimatized over 5 days (2–3 hours acclimatization to experiment room, 2 minutes handling, and 2 minutes loose-restraining with a towel). On experiment days, mice were moved to the experiment room 2 hours prior to the start of the experiment.

### Calibrated forceps

A commercially available algometer (Rodent Pincher-analgesia meter, Bioseb, Pinellas Park, USA) using calibrated forceps (11.25 cm long, one arm has a flattened end and the other arm has a smooth slug (φ = 3.5 mm) attached to the end) was used.

To measure the threshold for behavioral response to a noxious mechanical stimulus, a mouse was placed on a bench, had their head and body covered with a towel, and was loosely restrained. The tail was positioned between the forceps (2 cm from the tip of the tail) and force was applied gradually by hand at a constant rate (200 g every 2 seconds) until the nociceptive behavioral response occurred. Measurement of the nociceptive threshold was repeated 5 times with intervals of approximately 5 seconds. The maximum and minimum values were excluded from the sets of 5 measurements, and the residual three values were averaged to determine the threshold.

### Definition of behavioral responses

The following three types of behavioral responses were typically observed following pressure application: tail flicking, tail withdrawing, and struggling. Tail flicking was defined by the mouse’s tail quickly flicking upwards, followed by a sustained elevation for several seconds. Tail withdrawing was defined by the mouse wiggling its tail in an attempt to free it from the forceps, often resulting in the tail forming an S-like shape. Struggling, on the other hand, was defined by the mouse moving its body in an attempt to free its tail from the forceps. We determined this to be a nocifensive behavior rather than simply a response to restraint because acclimatization, mice very rarely struggled until pressure was applied to the tail. Tail withdrawing sometimes followed struggling and vice versa, but only the first response was recorded.

### Drug application

Remifentanil (Ultiva) was purchased from Janssen Japan. It was dissolved in saline at 10 μg/mL and injected subcutaneously (100 μg/kg) in the neck. In control mice, saline was injected subcutaneously.

### Comparison of response threshold among body parts

To compare the pain response threshold among body parts, we measured the threshold of the distal part (2 cm from the tip) and the proximal part (1cm from the base) of the tail and the left hind paw. During the loose-restraining session of acclimatization, we gently touched and tapped the tail and the left hind paw. We removed two mice from further measurement because they failed to become acclimatized to the gentle touching of their hind paw. Because the nociceptive threshold of the distal tail was stable during repeated measurements over 5 days, we measured the threshold for distal tail at day 1, proximal tail at day 2, and hind paw at day 3.

### Statistical analysis

All statistical analyses were performed with GraphPad Prism 6 (GraphPad Software, Inc). The criterion for statistical significance was *p* < 0.05 in all cases.

## Results

### Nociceptive response patterns evoked by calibrated forceps

**Fig 1:**
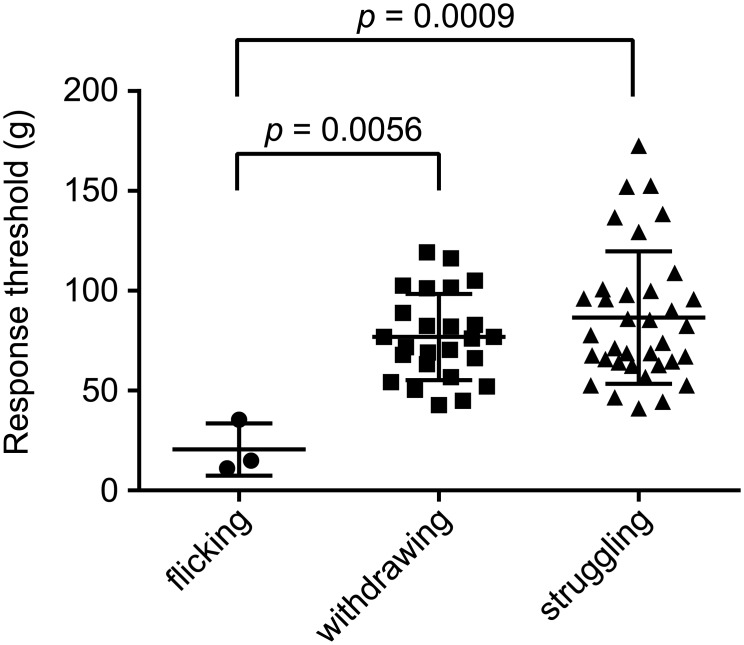
Three types of nociceptive behavior evoked by calibrated forceps. The response thresholds for the three types of nociceptive responses were plotted. One-way ANOVA with Tukey’s multiple comparison test revealed that the threshold of tail flicking was significantly lower than that of withdrawing (*p* = 0.0056) or struggling (*p* = 0.0009). The frequency of the tail flicking response was significantly lower than the other responses (3 out of 63 responses from 6 mice, *p* < 0.001, χ-squared test). Bars indicate mean ± SEM.

To evaluate the calibrated forceps for measuring the nociceptive response threshold to noxious mechanical stimuli in mice, we first examined the nociceptive response patterns evoked by pressure application to the tail. Under our experimental conditions, we observed three types of nociceptive behaviors: tail flicking, tail withdrawing, and struggling (*see* Definition of behavioral responses in Materials and methods). Vocalization was not observed during measurements. Fig 1 plots the distribution of nociceptive thresholds for each behavioral response. The threshold for tail flicking was significantly lower than tail withdrawing or struggling. Also, the frequency of tail flicking was significantly lower than the other responses. As a result, we decided to employ only the response thresholds for tail withdrawing and struggling for further analyses.

### Effect of acclimatization on the pincher test

**Fig 2:**
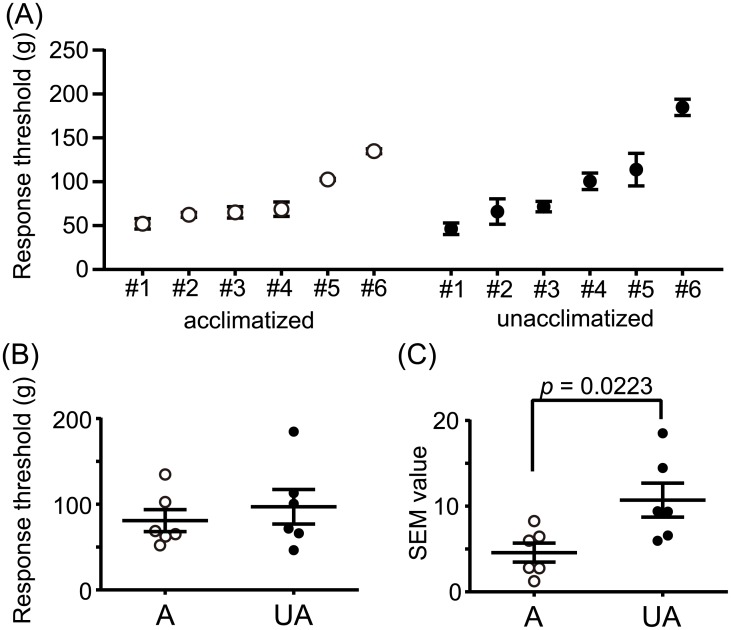
Effects of acclimatization on the nociceptive response threshold evoked by calibrated forceps. (A) The threshold distribution of acclimatized (open circles) and unacclimatized (filled circles) mice (*n* = 6, respectively) were plotted. (B) The mean values of the threshold were replotted. The distribution was not significantly different between the two groups (*t* = 0.6747, df = 10, *p* = 0.5152, unpaired t test). (C) The SEM values of the thresholds were replotted. The value was significantly lower in acclimatized mice (*t* = 2.700, df = 10, *p* = 0.0223, unpaired t test). Bars indicate mean ± SEM. A, acclimatized group; UA, unacclimatized group

To evaluate the effect of acclimatization on the measurement of the mechanical nociception threshold using calibrated forceps, we compared the distribution of the threshold between acclimatized and non-acclimatized mice. Fig 2A shows mean ± SEM of the threshold in acclimatized and unacclimatized mice. Though the mean values of the threshold were not significantly different between the two groups (Fig 2B), the SEM values were significantly lower in acclimatized mice (Fig 2C). These data conclude that our acclimatization protocols increase the precision of the threshold values obtained from the limited number of repeated measurements.

### Stable nociceptive threshold under repeated measurements over 5 days

**Fig 3:**
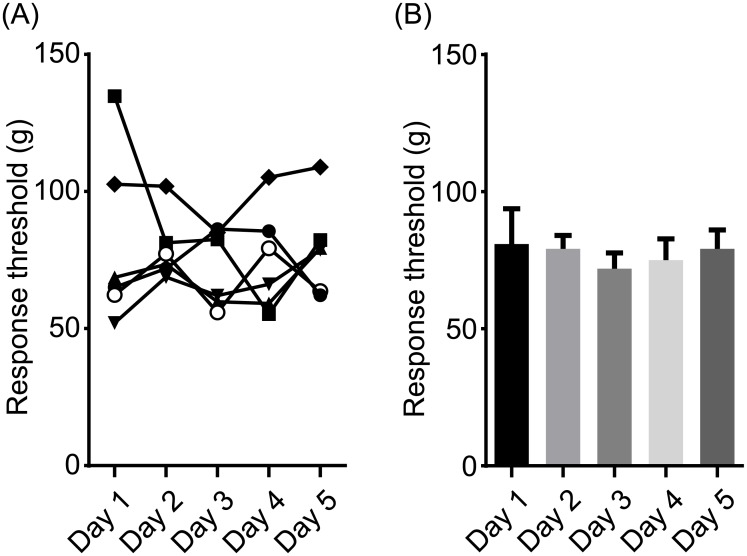
The threshold for nociceptive response measured by calibrated forceps was stable for 5 days. (A) Time courses of the nociceptive response threshold of 6 mice over 5 days. A repeated-measured one-way ANOVA revealed that there was no significant difference among days (F_4,20_ = 0.3240, *p* = 0.8585). (B) The thresholds were replotted as the mean ± SEM for 6 animals for each day.

Next, we measured the threshold daily for 5 consecutive days to examine the effect of repeated measurements. Fig 3 shows the time course of the threshold during the 5 days. There were no significant changes in the threshold. These data conclude that the response threshold to application of pressure to the tail was stable for a minimum of 5 days without any carryover effects.

### Evaluation of the calibrated forceps for measuring the rapid change of the threshold

**Fig 4:**
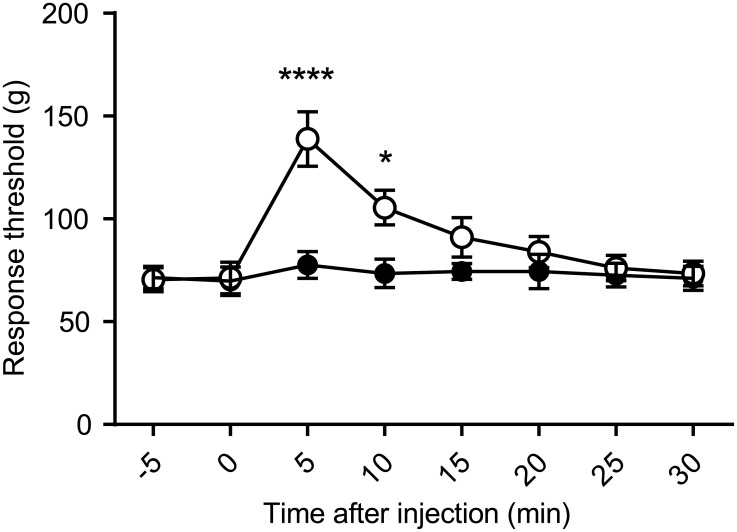
Rapid change in the mechanical nociception threshold induced by remifentanil treatment. Traces show the time course of the threshold of eight consecutive measurements with 5 minute intervals. Time 0 indicates the subcutaneous injection of remifentanil (open circle) or saline (filled circle). A repeated measures two-way ANOVA with Bonferroni multiple comparison test indicated that the threshold significantly increased at 5 and 10 minutes after remifentanil injection (**** indicated *p* < 0.0001, * indicated *p* = 0.0252). Data are represented as mean ± SEM. *n* = 6 for each group.

To examine whether the calibrated forceps were able to detect rapid changes in the mechanical nociceptive threshold, we measured the threshold every 5 minutes following a subcutaneous injection of remifentanil (100 μg/kg), a short-acting analgesic. Fig 4 shows the time courses of the thresholds. The threshold was significantly different between the remifentanil-treated group and the control group. A post-hoc Sidak’s multiple comparison test indicated that the threshold significantly increased at the 5 and 10 minute time points after remifentanil injection. Our data indicate that the calibrated forceps are suitable to assess a rapid change in the mechanical nociceptive threshold that may be induced by a short-acting analgesic.

### Comparison of the threshold at the distal part of the tail, the proximal part of the tail, and the hind paw using the calibrated forceps

**Fig 5:**
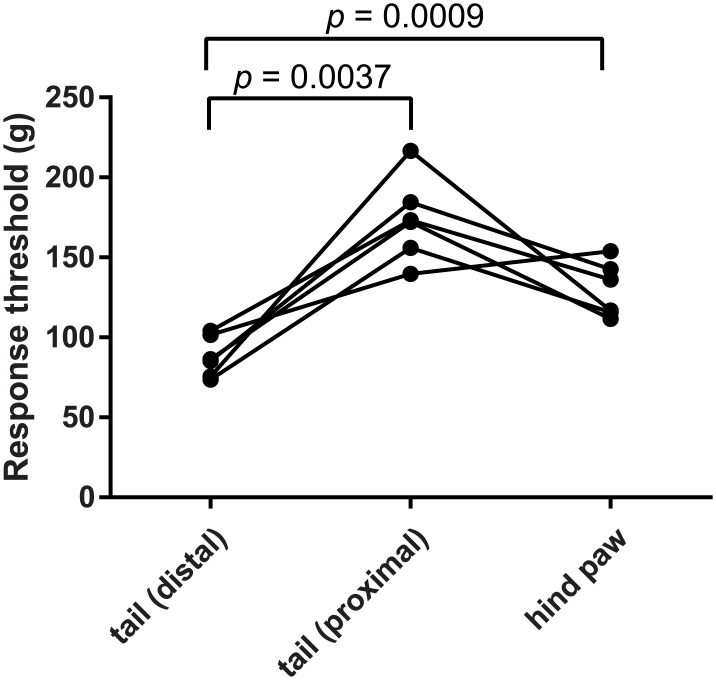
The distal part of the tail showed lower mechanical nociceptive threshold than the proximal part of the tail or the hind paw. Plots show the distribution of the threshold among the 3 body parts (distal tail, proximal tail, and hind paw) from six animals. A repeated-measures one-way ANOVA with Tukey’s multiple comparison test indicated that the threshold of the distal tail was significantly lower than that of the proximal tail or the hind paw (*** indicated *p* = 0.0009, ** indicated *p* = 0.0037). Note that the threshold of hind paw tended to be lower than that of the proximal tail (*p* = 0.071).

To examine the difference in the mechanical nociceptive threshold of body parts generally favored for performing pain tests, we measured and compared the threshold of the distal part of the tail, the proximal part of the tail, and the hind paw using calibrated forceps. Fig 5 shows the distribution of the threshold at the three body parts. The threshold was significantly different among the body parts. A post-hoc Tukey’s multiple comparison test indicated that the threshold of the distal part of tail was significantly lower than that of the proximal part of the tail or the hind paw.

## Discussion

In our preliminary trials, we originally planned to measure the nociceptive threshold in the hind paws of mice. However, even though the mice were acclimatized for 5 days, they always buried their hind paws beneath their bodies during the repetitive measurements required for high time resolution. In order to get a measurement, the hind paw needed to be forcibly pulled out, often causing a vocalization that suggested high stress. Therefore, we switched to measuring the nociceptive threshold in the tail for high time-resolution. It should be noted that we were able to measure the steady-state threshold of the mouse hind paw (Fig 5) as previously reported [19–21]. To apply the calibrated forceps to the hind paws of mice in a way that is necessary for a high time resolution, further refinement of acclimatization protocols or of holding procedures may be necessary.

Repeated daily measurements of the nociceptive threshold with calibrated forceps over 5 days resulted in stable and reliable values (Fig 3). These data suggest that the calibrated forceps could be used for repeated assessments within a few days without any carryover effects. Furthermore, the device may be useful for assessing the tail neuropathic pain by the combination of surgical injury of spinal nerve innervating the tail [24, 25].

The calibrated forceps also traced the rapid change of the mechanical nociceptive threshold induced by a short-acting analgesic (Fig 4). In the past, rapid changes in the nociceptive threshold have been examined by thermal tests. For example, the radiant heat test was used to examine the time course of antinociceptive effects caused by remifentanil in high time resolution (< 15 minutes) [26–28]. Our data suggest that the calibrated forceps may enable us to compare the effects of short-acting analgesics on the time course of mechanical nociception to the time course of thermal nociception.

One of the technical limitations in this study was the requirement of loose-restraint during measurement which is unnecessary in the von Frey test. It is well established that the 1 hour restraint stress evoked by immobilization in a restraining device induces stress-induced analgesia [29–31]. Although our restraining method could be considered as loose because the animals were able to move easily under the towel, we could not completely rule out the effect of stress.

In summary, our data demonstrate that the calibrated forceps are a stable and reliable tool to measure the mechanical nociceptive threshold at steady-state in mouse tail. Furthermore, the calibrated forceps were also well-suited to assess the rapid change in the mechanical nociceptive threshold caused by Remifentanil with 5 minute intervals which suggests that it may be useful for characterizing other short-acting analgesics as well.
